# Antioxidant Activity and Oxidative Stress-Oriented Apoptosis Pathway in Saccharides Supplemented Cryopreserved Sperm of Pacific Abalone, *Haliotis discus hannai*

**DOI:** 10.3390/antiox11071303

**Published:** 2022-06-29

**Authors:** Shaharior Hossen, Zahid Parvez Sukhan, Yusin Cho, Won Kyo Lee, Kang Hee Kho

**Affiliations:** Department of Fisheries Science, College of Fisheries and Ocean Sciences, Chonnam National University, 50 Daehak-ro, Yeosu 59626, Korea; shaharior@jnu.ac.kr (S.H.); sukhan1026@jnu.ac.kr (Z.P.S.); 198099@jnu.ac.kr (Y.C.); wklee196@jnu.ac.kr (W.K.L.)

**Keywords:** apoptosis, antioxidants, mRNA expression, O_2_^•−^ production, lipid peroxidation, DNA integrity, cryopreservation, Pacific abalone

## Abstract

The Pacific abalone *Haliotis discus hannai* is a highly commercialized seafood in Southeast Asia. The aim of the present study was to determine the antioxidant activity and oxidative stress-oriented apoptosis pathway in saccharides supplemented cryopreserved sperm of Pacific abalone. Cryopreserved sperm showed impaired antioxidant defenses due to the reduced mRNA abundance of antioxidant genes (CAT, Cu/Zn-SOD, Mn-SOD, GPx, GR, and BCL-2), apoptosis inhibitor (HSP70, and HSP90) gene, and enzymatic antioxidant activity compared to fresh sperm. Such impaired antioxidant defenses caused an increase in the mRNA expression of apoptosis genes (Bax, and Caspase-3), finally leading to apoptosis. The impaired antioxidant defense also increased O_2_^•−^ production and lipid peroxidation (MDA) levels, which further accelerated apoptosis. Considering all the experimental findings, an apoptosis pathway of cryopreserved sperm has been adopted for the first time. Specifically, sperm cryopreserved using 3% sucrose combined with 8% dimethyl sulfoxide (DMSO) showed improved mRNA stability, enzymatic activity, and DNA integrity with reduced O_2_^•−^ production and MDA levels compared to sperm cryopreserved with the other types of examined cryoprotectants (8% ethylene glycol + 1% glucose, 6% propylene glycol + 2% glucose, 2% glycerol + 3% glucose, and 2% methanol + 4% trehalose). The present study suggests that 3% sucrose combined with 8% DMSO is suitable to cryopreserve the sperm of this valuable species for molecular conservation.

## 1. Introduction

Pacific abalone, *Haliotis discus hannai* is high-demandable and high-priced seafood because it contains antioxidant- and anticancer-activated bioactive molecules [1,2]. In South Korea, it is known as the “emperor of shellfish” [3], and worldwide known as “soft gold” of the Ocean [4]. Commercial fertilization of Pacific abalone depends on broodstock conditioning through effective accumulating temperature (EAT). A large number of male abalone needed to maintain EAT [2]. Cryopreserved sperm can solve this issue by supplying sperm during in vitro fertilization. Further, sperm cryopreservation can be used to preserve genetic materials of this important species. Sperm cryopreservation biotechnology has several benefits including genetic materials preservation, maintenance costs reduction of male broodstock, constant supply, simplified transportation and to reduce disease transfer [5,6,7].

Despite its benefits, the freeze–thaw process of cryopreservation is known to generate excessive reactive oxygen species (ROS) [8]. Excessive accumulation of ROS leads to oxidative stress and reduce the motility, viability, and enzymatic activity of sperm [9]. Oxidative stress can also stimulate lipid peroxidation (LPO) and DNA fragmentation [10]. Sperm plasma membrane contains highly poly unsaturated fatty acids that are prone to oxidation by ROS or other free radicals. This oxidation can increase the level of membrane LPO that can be detected using malondialdehyde (MDA) indicator [9]. Antioxidant enzymes can counteract ROS production in cryopreservation [11]. Antioxidant enzymes are categorized into five major types: catalase (CAT), Cu/Zn-superoxide dismutase (Cu/Zn-SOD), manganese superoxide dismutase (Mn-SOD), glutathione peroxidase (GPx), and glutathione reductase (GR) [12]. Cryopreservation can affect the antioxidant defense system [8].

Cryopreservation process can also reduce mRNA expression levels of five vital antioxidant enzymes (CAT, Cu/Zn-SOD, Mn-SOD, GPx, and GR) in mammalian sperm [13,14,15]. Imbalance in antioxidant defense might be responsible for mitochondria-oriented apoptosis [16,17,18].

Cryopreservation can trigger the apoptosis mechanism in post-thaw sperm [19,20]. Apoptosis is a physiological and biological process that is crucial for normal development and homeostasis maintenance [21,22]. Apoptosis is largely regulated by the activation of a class of cysteine proteases called caspases, and apoptosis deregulation can lead to infertile sperm [23]. The vital characteristics of apoptosis include cell wrinkling, DNA damage, chromatin compaction, and membrane destruction [21,24]. B-cell lymphoma 2 (BCL-2) suppresses apoptosis, whereas BCL-2-associated X (Bax) promotes apoptosis [21]. BCL-2 can also maintain the mitochondrial membrane potential (MMP) through a receptor binding mechanism with the outer mitochondrial membrane [25].

Information about oxidative stress-oriented apoptosis pathway of post thaw sperm of Pacific abalone is currently unavailable. There have not any information about antioxidant activity, ROS production, and LPO in cryopreserved sperm of Pacific abalone. Further, to the best of our knowledge, no information has been reported about apoptosis and antioxidant gene expression of cryopreserved sperm of Pacific abalone, fish, or aquatic invertebrates. Hence, the aim of the present study was to determine antioxidant activity and oxidative stress-oriented apoptosis pathway in saccharides supplemented cryopreserved sperm of Pacific abalone. Oxidative stress pathway was presented based on quantitative real-time PCR (qRT-PCR), enzymatic activities, plasma membrane integrity (PMI), MMP, O_2_^•−^ production, MDA content, and DNA fragmentation data. qRT-PCR was performed to quantify mRNA abundance of apoptosis associated genes (Bax, BCL-2, Caspase-3, HSP70, and HSP90), and antioxidant genes (CAT, Cu/Zn-SOD, Mn-SOD, GPx, and GR). Enzymatic activities (CAT, SOD, and GSH), and LPO activities (MDA content) were measured using a microplate reader. Fluorescent technique was applied to determined PMI, MMP, O_2_^•−^, and DNA integrity of saccharide supplemented cryopreserved sperm of Pacific abalone.

## 2. Materials and Methods

### 2.1. Abalone Handling, Management, and Sperm Collection

Three years old biologically matured Pacific abalone (*H. discus hannai*) were collected from a commercial abalone hatchery (Tou-Jong Soosan, Yeosu, Korea) during the peak breeding season. Abalone were reared in continuous seawater and aeration facilitated cemented tanks. Sugar kelp, *Saccharina latissimi* was supplied as food for abalone. Experimental abalone were chosen based on the criteria described previously by the same research group [26].

A total of 160 abalone with an average body weight of 135.7 ± 5.3 g and shell length of 107.41 ± 4.6 mm were used to conduct the experiments. Before inducing, abalone were tissue-toweled to avoid water contamination with sperm. A heat-inducing method was applied to induce the abalone for spawning by previously described method [2], with minor modification. Briefly, abalone were sun dried for 45 min with the shell facing down and another 20 min with the muscle facing down. Sperm were collected by gently stripping the induced abalone. Sperm were immediately collected in Eppendorf tubes and kept in a refrigerator (4 °C) until used.

### 2.2. Motility Observation of Fresh Sperm

Sperm motility was observed based on visual estimates and recorded according to previously established method for the same species [2,3,26]. Briefly, 10 µL sperm were diluted with 100 µL of filtered seawater (FSW). Subsequently, 2 µL of diluted sperm was mixed with 100 µL of FSW on a glass slide and visualized under a microscope (Nikon eclipse E200, Tokyo, Japan) using 20× objective lens. Sperm motility was calculated ten times using ten different samples. Sperm having over 90% motility were considered for cryopreservation experiments of Pacific abalone.

### 2.3. Experimental Reagents

All experimental reagents were purchased from Sigma-Aldrich (Saint Louis, MO, USA), unless mentioned otherwise.

### 2.4. Sperm Cryopreservation Protocol

In this experiment, saccharides (sucrose, glucose or trehalose) were used in combination with previously recommended penetrating cryoprotectant (P-CPA: 8% DMSO, 8% EG, 6% PG, 2% GLY, and 2% MeOH) by the same research group [2,3]. Saccharides at different concentrations (1, 2, 3, 4, and 5%) were selected based on the findings of Liu et al. [27]. A preliminary experiment was conducted to find out suitable concentration and combination of saccharides with each of the P-CPA (data not shown). Considering the improved post-thaw motility of preliminary experiments, present study recommended five types of cryoprotectant solution including 8% DMSO + 3% sucrose, 8% EG + 1% glucose, 6% PG + 2% glucose, 2% GLY + 3% glucose and 2% MeOH + 4% trehalose for farther cryopreservation experiments.

Sperm were diluted with FSW at a ratio of 1:10. Cryoprotectant solution was prepared by mixing P-CPA (8% DMSO, 8% EG, 6% PG, 2% GLY, and 2% MeOH) with non-penetrating CPA (glucose, sucrose, or trehalose). Briefly, 8% DMSO, 8% EG, 6% PG, 2% GLY, and 2% MeOH solution of penetrating CPA were prepared according to method described previously [2,3]. Saccharides were then supplemented with penetrating CPA solutions as combinations of 8% DMSO + 3% sucrose, 8% EG + 1% glucose, 6% PG + 2% glucose, 2% GLY + 3% glucose and 2% MeOH + 4% trehalose. Diluted sperm were properly mixed with final cryoprotectant solution at an equal ratio. Sperm were equilibrated for 10 min and transferred into 0.5 mL straws using an Eppendorf syringe. Straws were sealed with powder sealer and placed on 5 cm rack height of a Styrofoam box for 10 min and then submerged into liquid nitrogen (LN). Sperm in straws were stored in a 38 L LN tank (model: 38VHC-11M, serial: 80907, Worthington Industries, Hamilton, NY, USA) until further use. Straws were thawed at 60 °C in a water bath (JISICO Lab & Scientific Instrument, Seoul, Korea). As control, sperm were also cryopreserved using P-CPA (8% DMSO, 8% EG, 6% PG, 2% GLY, and 2% MeOH) according to method described by Hossen et al. [2]. Post-thaw motility was detected according to method described in previous Section 2.2.

### 2.5. Detection of PMI and MMP of Saccharide Supplemented Cryopreserved Sperm of Pacific Abalone

Plasma membrane integrity (PMI) of fresh and saccharide supplemented cryopreserved sperm samples were evaluated using a LIVE/DEAD^®^ sperm viability kit (Invitrogen Molecular Probes, Eugene, OR, USA) according to previously described protocol [2], with slight modifications. Briefly, sample were diluted with FSW at a final concentration of 1.8 × 10^6^ cells/mL. An aliquot of SYBR^TM^ 14 dye (5 µL) and propidium iodide (PI: 10 µL) were gently mixed with 1000 μL of samples and incubated at 37 °C for 15 min in the dark. An aliquot of SYBR14/PI-stained sample was placed on a slide glass and visualized under a fluorescence microscope (Eclipse E600, Nikon, Tokyo, Japan). SYBR14-stained images of intact plasma membrane were captured using a green filter (wavelength: 450–490 nm) and PI-stained images of damaged plasma membranes were captured using a red filter (wavelength: 510–560 nm). The fluorescence images captured with the green and red filters were merged with pictures taken without a filter to detect the PMI of fresh and saccharide supplemented cryo-preserved sperm. The visualization and analysis (*n* = 10) were performed according to the method described by Hossen et al. [7].

The mitochondrial membrane potential (MMP) of fresh and saccharide supplemented cryopreserved sperm samples were evaluated using previously described Rh123/PI^®^ method [2], with minor modification. Briefly, an aliquot of 0.8 μL of Rh123 and 10 μL of PI were mixed with 1000 μL of sample (1.8 × 10^6^ cells/mL) and incubated at 20 °C in the dark for 20 min. Rh123-stained images of intact mitochondrial membranes were captured using a green filter (450–490 nm) and PI-stained images of damaged mitochondrial membranes were taken using a red filter (510–560 nm). The fluorescence images captured with the green and red filters were merged with pictures taken without a filter to detect the MMP of fresh and saccharide supplemented cryopreserved sperm. The visualization and analysis (*n* = 10) were performed according to the method described for PMI.

### 2.6. Production of Superoxide Anion (O_2_^•−^)

Production of superoxide anion (O_2_^•−^) by fresh and cryopreserved sperm were detected using DHE/SYTOX^®^ green kit according to a previously published protocol [28], with slight modifications. Briefly, sperm were diluted with 250 μL FSW to have a final concentration of 1 × 10^6^ cells/mL. Sample was stained with 3 μL DHE (5 mM) and 1 μL SYTOX (5 mM). Samples were incubated in a cool block (model: ALB6400, FINEPCR^®^, Gunpo, Korea) at 10 °C for 10 min in the dark. Subsequently, a sperm sample was visualized under a fluorescent microscope (excitation: 450–490 nm, emission: 510–560 nm, eclipse E600, Nikon, Tokyo, Japan). Ten replications (*n* = 10) were considered to analyze the (O_2_^•−^) values, and a minimum of 200 sperm cells were considered in each replication.

### 2.7. Comet Assay

Comet assay of saccharide supplemented cryopreserved sperm were performed using a Comet assay^®^ kit according to a previously published protocol [2], with slight modifications. Sperm (1 × 10^5^ cells/mL) were immobilized on comet slides^TM^ using comet agarose (supplemented with kit). Samples were treated with pre-chilled (4 °C) lysis buffer solution for one hour and then treated with pre-chilled (4 °C) alkaline unwinding solution (newly prepared) for another one hour. After that, slides were electrophoresed in a cometAssay^®^ electrophoresis system for 30 min at 21 V with an alkaline electrophoresis solution. Slides were washed (5 min) twice with deionized distilled water, dried for 30 min in a 37 °C incubator, and stained with Vista green DNA dye. Stained comets were visualized and captured using a fluorescence microscope (450–490 nm, Nikon eclipse E600, Tokyo, Japan) with 20× lens. A minimum of 200 comets from each treatment (*n* = 5) were used to analyze comet parameters. Several parameters such as tail length (μ), DNA integrity, DNA fragmentation, and tail moment (μ) of comets were analyzed using a comet Assay IV image analysis software (version 4.3.2, Perceptive Instruments Ltd., Bury Saint Edmunds, UK).

### 2.8. Measurements of Antioxidant Enzyme Activities

#### 2.8.1. Catalase (CAT) Activity

Fresh and cryopreserved sperm samples (3.87 × 10^6^ cells/mL) were centrifuged at 8000× *g* for 5 min at 4 °C to obtain sperm pellets. These sperm pellets were resuspended in ice-cold PBS and sonicated for 30 s using a sonicator (CL-188, Qsonica, Church Hill Rd, Newtown, CT, USA). After centrifuging each sample at 10,000× *g* for 15 min at 4 °C (Microfuge^®^ 20R centrifuge, Beckman Coulter, CA, USA), the supernatant was split into micro tubes. Sample was immediately used to detect catalase activity using a catalase colorimetric activity kit (Catalog# EIACATC, Invitrogen™, Frederick, MD, USA) according to the manufacturer’s instructions. The catalase concentration (U/mL) (*n* = 10) at 560 nm were measured using an Epoch^TM^ Microplate Spectrophotometer (Epoch 2, BioTek, Winooski, VT, USA).

#### 2.8.2. Superoxide Dismutase (SOD) Activity

Superoxide dismutase (SOD) activity of cryopreserved sperm was measured using a SOD colorimetric activity kit (Catalog# EIASODC, Invitrogen, Frederick, USA). Fresh and cryopreserved sperm samples (3.87 × 10^6^ cells/mL) were centrifuged at 8000× *g* for 5 min at 4 °C to obtain sperm pellets. These sperm pellets were resuspended with PBS and sonicated for 30 s. Samples were centrifuged at 1500× *g* for 10 min at 4 °C to collect the supernatant. SOD activity assay (*n* = 10) was performed using the supernatant according to the manufacturer’s instructions. SOD concentration (U/mL) was measured at 450 nm using an Epoch^TM^ Microplate Spectrophotometer (Epoch 2, BioTek, Winooski, VT, USA).

#### 2.8.3. Glutathione (GSH) Activity

The glutathione (GSH) activity of cryopreserved sperm was measured using a glutathione colorimetric detection kit (catalog # EIAGSHC, Invitrogen™, Frederick, MD, USA). Briefly, fresh and cryopreserved sperm samples (3.87 × 10^6^ cells/mL) were centrifuged at 8000× *g* for 5 min at 4 °C to obtain sperm pellets. The samples were homogenized with 5% SSA (aqueous 5-sulfosalicylic acid) in ice-cold PBS and incubated at 4 °C for 10 min. Homogenized samples were then centrifuged at 14,000 rpm for 10 min at 4 °C to collect the supernatant for the GSH assay. The GSH assay (*n* = 10) was performed using a glutathione colorimetric detection kit (catalog # EIAGSHC, Invitrogen™, Frederick, MD, USA) according to the manufacturer’s protocol. Lastly, glutathione activity (U/mL) was measured at 405 nm using an Epoch^TM^ microplate spectrophotometer (Epoch 2, BioTek, Winooski, VT, USA).

### 2.9. Lipid Peroxidation (MDA) Assay

Lipid peroxidation (MDA) levels in fresh and cryopreserved sperm (3.87 × 10^6^ cells/mL) were detected using a lipid peroxidation (MDA) assay kit (catalog # K739-100, BioVision, CA, USA) following the manufacturer’s protocol. Briefly, samples were homogenized using MDA lysis buffer and centrifuged at 13,000× *g* for 10 min at 4 °C. The supernatant (200 μL) was incubated with 600 μL of TBA buffer for 60 min at 95 °C. In the next step, the samples were incubated in an ice bath for 10 min, and 1-butanol (300 μL) and 5 M NaCl (100 μL) were added to the sample, which was placed on a vortex mixer. The samples were then centrifuged at 16,000× *g* for 3 min at 20 °C. The supernatant was collected and heated to 55 °C to evaporate the n-butanol. The residue was then resuspended in ultrapure water. Subsequently, a 200 μL sample (*n* = 10) was used to detect the absorbance of MDA at 532 nm with a microplate reader.

### 2.10. Total RNA (tRNAs) Extraction and cDNA Synthesis of Cryopreserved Sperm

Total RNA (tRNAs) was extracted from fresh sperm and cryopreserved sperm samples using a RNeasy mini kit (Qiagen, Hilden, Germany) according to the method described previously [2,26]. RNase-free DNase (Promega, Madison, WI, USA) treatment was performed to eliminate genomic DNA contamination. A Superscript^®^ III First-Strand synthesis kit (Invitrogen, Carlsbad, CA, USA) was used to reverse transcribed tRNAs to cDNAs. tRNA and cDNA concentrations were measured with a spectrophotometer (ACTGene ASP-2680, Piscataway, USA).

### 2.11. Quantitative Real-Time PCR (qPCR)

qRT-PCR was conducted to determine the mRNA expression levels of five vital antioxidant gene (CAT, Cu/Zn-SOD, Mn-SOD, GPx, and GR) and apoptosis associated gene (Caspase-3, Bax, BCL-2, HSP70, and HSP90) in fresh and cryopreserved sperm samples. Gene-specific primers (Table 1) were used to conduct qRT-PCR in a LightCycler^®^ 96 System (Roche, Germany) following recommended methods [2,26]. Briefly, a reaction mix (20 µL) was prepared by using SyGreen Mix (10 µL), each forward and reverse primer (1 µL), cDNA template (1 µL), and PCR-grade water (7 µL) to perform qRT-PCR. PCR condition was fixed according to described by Hossen et al. (2021a). The mRNA expression levels were quantified following the 2^−ΔΔct^ method [29]. A house keeping β-actin gene was used to normalize the expression levels.

### 2.12. Apoptosis Pathway of Cryopreserved Pacific Abalone Sperm

The apoptosis pathway of cryopreserved sperm was analyzed based on findings of apoptosis (Bax, and Caspase-3) and anti-apoptosis (BCL-2) gene expression, antioxidant gene expression (CAT, Cu/Zn-SOD, Mn-SOD, GPx and GR), HSPs gene expression (HSP70, and HSP90), enzymatic activity (CAT, Cu/Zn-SOD, Mn-SOD, GSH, and GR), MDA levels, DNA fragmentation, PMI, MMP, and O_2_^•−^ production.

### 2.13. Fertility and Hatchability Test

Three-years-old matured females (40) were used in fertilization experiment. Abalone were induced according to method described previously [2]. After spawning, egg quality was examined under a microscope (Nikon Eclipse E200, Tokyo, Japan). Based on the findings of previous sections, 8% DMSO + 3% S was used to conduct fertilization experiment. Sperm cryopreserved using 8% DMSO were used as control. Fertilization experiments were performed in a series of plastic bowls (2L). Then, 40,000 eggs were used in each treatment (*n* = 3). Sperm to egg ratios of 10,000:1 was maintained in this experiment according to recommended previously [2]. Experimental water temperature was maintained at 18–20 °C. Fertilization and hatching rate were calculated according to method described previously [2,26].

### 2.14. Statistical Analysis

All statistical analyses were performed using SPSS 26.00 (SPSS Inc., Chicago, IL, USA). All statistical data generated in the figures and tables are presented as the mean ± standard deviation (SD). One-way analysis of variance (ANOVA) and Duncan’s multiple comparisons test were used to evaluate differences between different treatments. Differences were considered statistically significant at *p* < 0.05. Pearson correlation analysis was performed to determine the relationships between sperm quality and oxidative stress-associated parameters. GraphPad Prism software (GraphPad Prism version 9.3.1 for Windows; GraphPad Software, San Diego, CA, USA) was used to generate the graphs. Pearson’s correlation coefficient was determined using a standard procedure in SPSS 26.00. Correlation was considered significant at the 0.01 level (two-tailed).

## 3. Results

### 3.1. Post-Thaw Sperm Quality

#### 3.1.1. Post-Thaw Motility of Saccharide Supplemented Sperm

**Figure 1:**
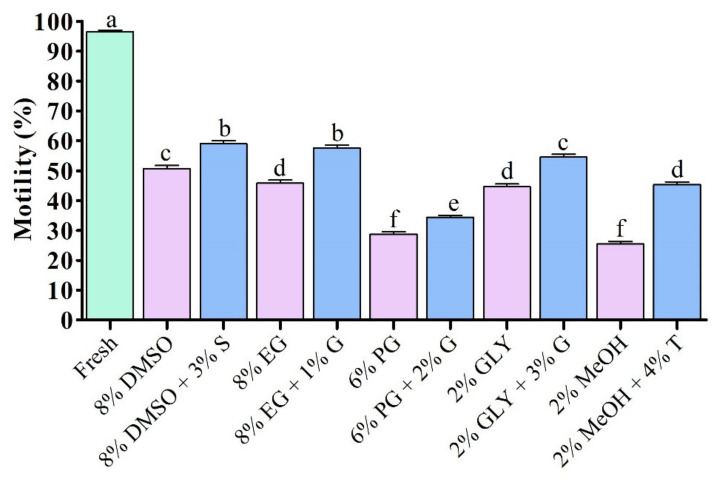
Post-thaw motility of saccharide supplemented cryopreserved sperm of Pacific abalone. DMSO: dimethyl sulfoxide, EG: ethylene glycol, PG: propylene glycol, GLY: glycerol, MeOH: methanol, S: sucrose, G: glucose, T: trehalose. Significantly different levels (*p* < 0.05) are denoted by different letters.

Post-thaw motility of sperm cryopreserved using saccharides are presented in Figure 1. Saccharides combined with P-CPA significantly improved post-thaw motility compared to sperm cryopreserved using P-CPA only (Figure 1). Sperm cryopreserved using 8% DMSO + 3% S or 8% EG + 1% G showed significantly improved post-thaw motility than those cryopreserved using other cryoprotectants (Figure 1).

#### 3.1.2. PMI and MMP of Saccharide Supplemented Cryopreserved Sperm

**Figure 2:**
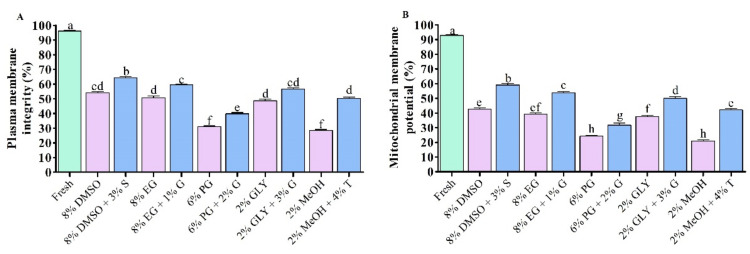
Post-thaw sperm quality of saccharide supplemented cryopreserved sperm of Pacific abalone. (**A**) Plasma membrane integrity (PMI) and (**B**) Mitochondrial membrane potential (MMP) of saccharide supplemented cryopreserved sperm. DMSO: dimethyl sulfoxide, EG: ethylene glycol, PG: propylene glycol, GLY: glycerol, MeOH: methanol, S: sucrose, G: glucose, T: trehalose. Significantly different levels (*p* < 0.05) are denoted by different letters.

Sperm cryopreserved using 8% DMSO + 3% S showed significantly improved PMI (Figure 2A), and MMP (Figure 2B) compared to sperm cryopreserved using other types of CPA. However, PMI and MMP of cryopreserved sperm were significantly lower (*p* < 0.05) compared to fresh sperm.

### 3.2. Production of Superoxide Anion (O_2_^•−^)

**Figure 3:**
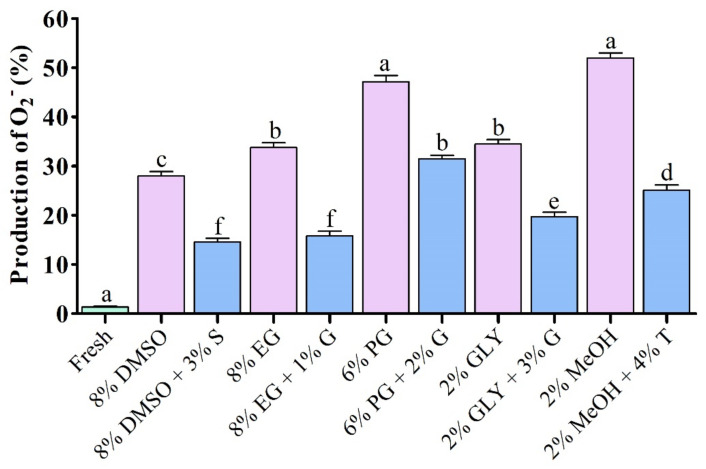
Superoxide anion production (O_2_^•−^) in different types of post-thaw sperm. Results are presented as mean values ± standard deviation (*n* = 10). DMSO: dimethyl sulfoxide, EG: ethylene glycol, PG: propylene glycol, GLY: glycerol, MeOH: methanol, S: sucrose, G: glucose, T: trehalose. Significantly different levels (*p* < 0.05) are denoted by different letters.

Sperm cryopreserved using saccharides showed lower production of superoxide anion (O_2_^•−^) than those cryopreserved without supplementation of saccharides (Figure 3). Sperm cryopreserved using 3% sucrose combined with 8% DMSO showed lower production of O_2_^•−^ (14.6 ± 2.4%) than those cryopreserved with other types of cryoprotectant solution (Figure 3). However, fresh sperm showed significantly (*p* < 0.05) lower production of O_2_^•−^ (0.9 ± 0.5%) than all types of post-thaw sperm.

### 3.3. Sperm DNA Integrity

**Figure 4:**
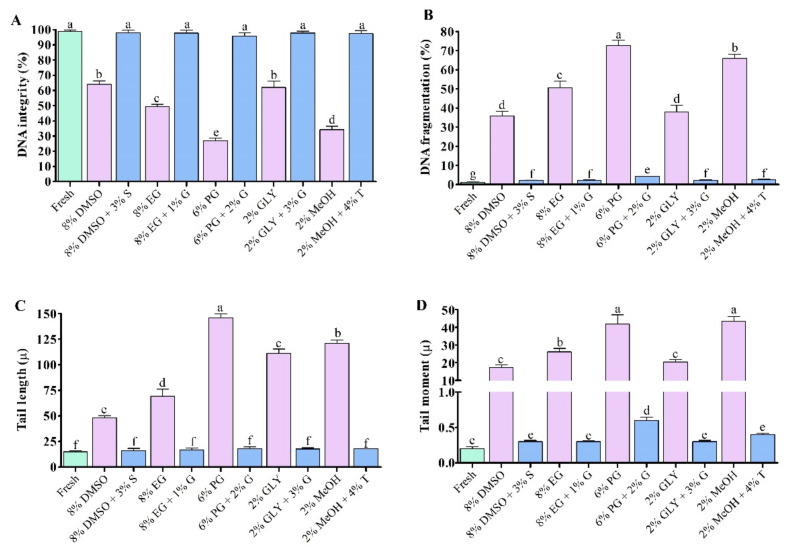
Comet assay parameters of sperm cryopreserved using different types of saccharides. (**A**) DNA integrity, (**B**) DNA fragmentation, (**C**) Tail length, and (**D**) Tail moment of sperm cryopreserved using different types of saccharides. Results are presented as mean values ± standard deviation (*n* = 5). DMSO: dimethyl sulfoxide, EG: ethylene glycol, PG: propylene glycol, GLY: glycerol, MeOH: methanol, S: sucrose, G: glucose, T: trehalose. Significantly different levels (*p* < 0.05) are denoted by different letters.

Results of comet assay parameters of sperm cryopreserved using saccharides are shown in Figure 4. Saccharide supplemented cryopreserved sperm showed dramatical improvement of DNA integrity compared to sperm cryopreserved without supplementation of saccharides (Figure 4). However, DNA integrity of sperm cryopreserved using saccharides did not show significant (*p* > 0.05) differences with fresh sperm (Figure 4A). DNA fragmentation, tail length and tail moment of sperm cryopreserved using 8% DMSO + 3% S was not significantly *(p* > 0.05) different from that of sperm cryo-preserved using EG + 1% G or using 2% GLY + 3% G (Figure 4B–D).

### 3.4. Antioxidant Enzyme Activities

#### 3.4.1. Catalase (CAT) Activity

**Figure 5:**
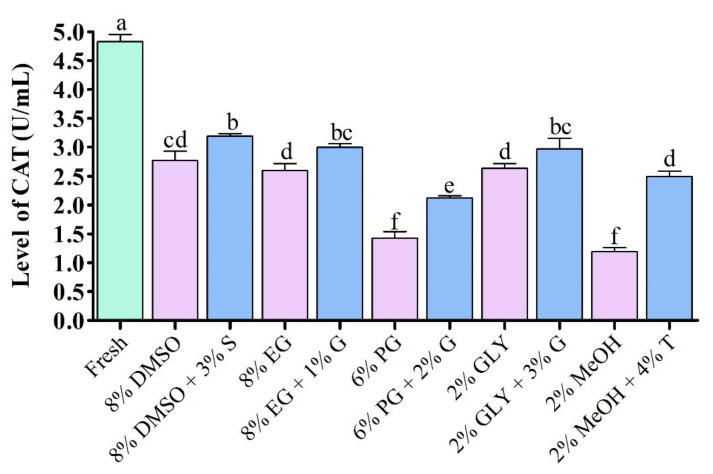
Enzymatic activities of catalase (CAT) in different types of post-thaw sperm cryopreserved using saccharides. DMSO: dimethyl sulfoxide, EG: ethylene glycol, PG: propylene glycol, GLY: glycerol, MeOH: methanol, S: sucrose, G: glucose, T: trehalose. Significantly different levels (*p* < 0.05) are denoted by different letters. Results are presented as mean values ± standard deviation (*n* = 10).

Catalase activities of fresh sperm and sperm cryopreserved using saccharides are given in Figure 5. Supplementation of saccharides during cryopreservation improved catalase (CAT) activity than the control without addition of saccharides (Figure 5). Cryopreserved sperm showed significantly (*p* < 0.05) reduced CAT activity compared to fresh sperm. However, sperm cryopreserved using 3% sucrose combined with 8% DMSO showed significantly higher (*p* < 0.05) CAT activity (3.1 ± 0.1 U/mL) than sperm cryopreserved using other types of cryoprotectant.

#### 3.4.2. Superoxide Dismutase (SOD) Activity

**Figure 6:**
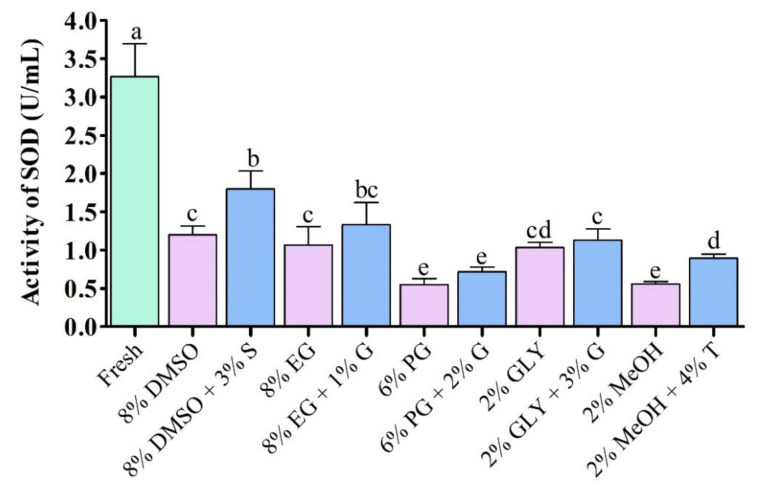
Enzymatic activities of superoxide dismutase (SOD) in different types of post-thaw sperm cryopreserved using saccharides. DMSO: dimethyl sulfoxide, EG: ethylene glycol, PG: propylene glycol, GLY: glycerol, MeOH: methanol, S: sucrose, G: glucose, T: trehalose. Results are presented as mean values ± standard deviation (*n* = 10). Significantly different levels (*p* < 0.05) are denoted by different letters.

Supplementation with saccharides during cryopreservation improved superoxide dismutase (SOD) activity compared to the control without addition of saccharides (Figure 6). Sperm cryopreserved using 3% sucrose combined with 8% DMSO showed significantly (*p* < 0.05) improved SOD activity (1.8 ± 0.4 U/mL) than those cryopreserved using other types of cryoprotectant (Figure 6). However, fresh sperm showed significantly (*p* < 0.05) higher SOD activity (3.3 ± 0.7 U/mL) than cryopreserved sperm.

#### 3.4.3. Glutathione (GSH) Activity

**Figure 7:**
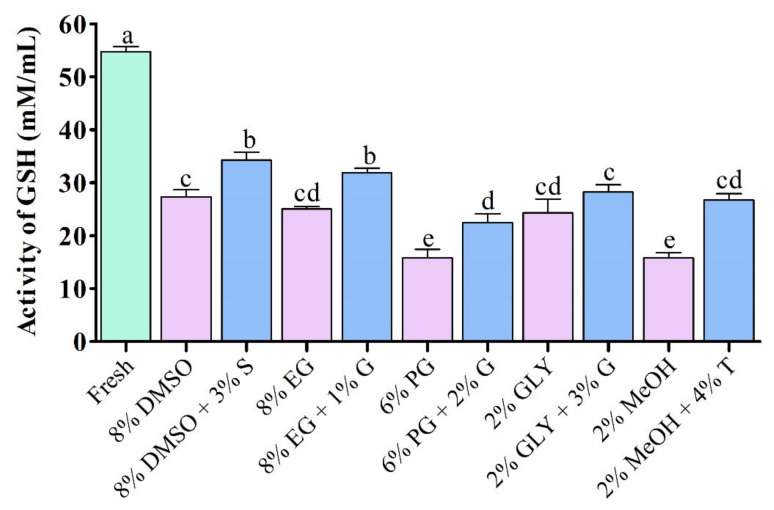
Enzymatic activities of glutathione (GSH) in different types of post-thaw sperm cryopreserved using saccharides. DMSO: dimethyl sulfoxide, EG: ethylene glycol, PG: propylene glycol, GLY: glycerol, MeOH: methanol, S: sucrose, G: glucose, T: trehalose. Results are presented as mean values ± standard deviation (*n* = 10). Significantly different levels (*p* < 0.05) are denoted by different letters.

Supplementation with saccharides during cryopreservation improved glutathione (GSH) activity than the control without addition of saccharides (Figure 7). GSH activities of fresh sperm and cryopreserved sperm are given in Figure 7. Sperm cryopreserved using 3% sucrose combined with 8% DMSO showed significantly (*p* < 0.05) improved GSH activity (34.3 ± 2.6 mM/mL) compared to sperm cryopreserved using other types of cryoprotectant (Figure 7). However, fresh sperm showed significantly (*p* < 0.05) higher GSH activity (54.8 ± 1.7 mM/mL) than cryopreserved sperm.

### 3.5. Lipid Peroxidation (MDA) Activity

**Figure 8:**
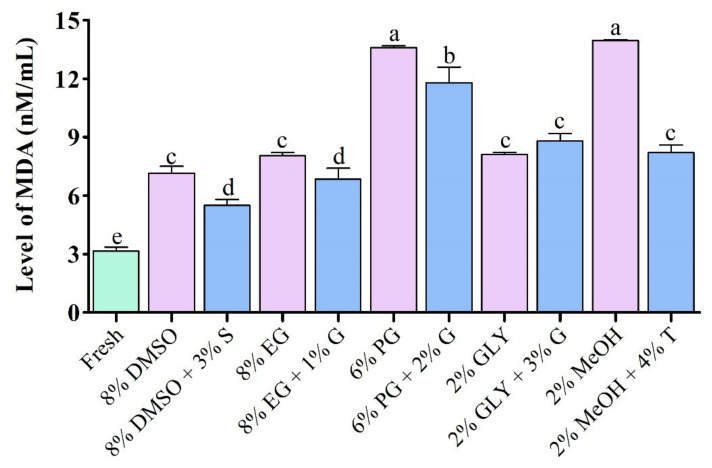
Lipid peroxidation (MDA) activities in different types of post-thaw sperm cryopreserved using saccharides. DMSO: dimethyl sulfoxide, EG: ethylene glycol, PG: propylene glycol, GLY: glycerol, MeOH: methanol, S: sucrose, G: glucose, T: trehalose. Results are presented as mean values ± standard deviation (*n* = 10). Significantly different levels (*p* < 0.05) are denoted by different letters.

Results of MDA measurement revealed that the cryopreservation process significantly (*p* < 0.05) induced lipid peroxidation activity (Figure 8). Sperm cryopreserved without addition of saccharides showed the highest levels of MDA (Figure 8). Overall, sperm cryopreserved using saccharides with CPA had lower levels of MDA (Figure 8). Sperm cryopreserved using 8% DMSO + 3% S showed a significantly lower level (5.5 ± 0.4 nM/mL) of MDA than other types of post-thaw sperm (Figure 8).

### 3.6. Expression Analysis of Vital Antioxidant Gene in Saccharide Supplimented Cryopreserved Sperm

#### 3.6.1. Expression Analysis of CAT mRNA Transcript

**Figure 9:**
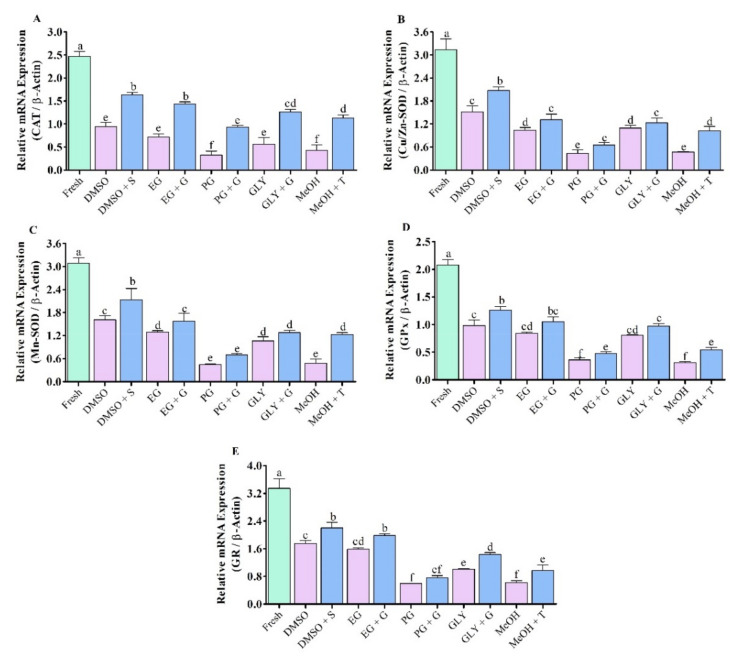
mRNA abundance of antioxidant gene in post-thaw Pacific abalone sperm cryopreserved using saccharides. (**A**) Catalase (CAT), (**B**) Copper/zinc superoxide dismutase (Cu/Zn-SOD, (**C**) Manganese superoxide dismutase (Mn-SOD), (**D**) Glutathione peroxidase (GPx), (**E**) Glutathione reductase (GR). Expression values of mRNA were normalized against average ΔCT values of control. DMSO: 8% dimethyl sulfoxide, EG: 8% ethylene glycol, PG: 6% propylene glycol, GLY: 2% glycerol, MeOH: 2% methanol, DMSO + S: 8% DMSO combined with 3% sucrose (S), EG + G: 8% EG combined with 1% glucose (G), PG + G: 6% PG combined with 2% glucose (G), GLY + G: 2% GLY combined with 3% glucose (G), MeOH + T: 2% MeOH combined with 4% trehalose (T). Significantly different levels (*p* < 0.05) are denoted by different letters.

Relative mRNA expression levels of CAT in fresh and cryopreserved sperm are given in Figure 9A. Saccharide supplemented cryopreserved sperm showed higher CAT mRNA transcript levels compared to sperm cryopreserved without supplementation of saccharides. CAT mRNA transcript levels in sperm cryopreserved using 3% sucrose combined with 8% DMSO were higher than those in other types of post-thaw sperm. However, CAT mRNA transcript levels of sperm cryopreserved using 3% sucrose combined with 8% DMSO was not significantly (*p* > 0.05) different from that of sperm cryopreserved using 1% glucose combined with 8% EG.

#### 3.6.2. Expression Analysis of Cu/Zn-SOD mRNA Transcript

Saccharide supplemented cryopreserved sperm showed higher Cu/Zn-SOD mRNA transcript levels compared to sperm cryopreserved without supplementation of saccharides. However, sperm cryopreserved using 3% sucrose combined with 8% DMSO showed significantly (*p* < 0.05) higher Cu/Zn-SOD mRNA transcript abundance compared to other types of post-thaw sperm (Figure 9B).

#### 3.6.3. Expression Analysis of Mn-SOD mRNA Transcript

Results of Mn-SOD mRNA expression in fresh and cryopreserved sperm are given in Figure 9C. All post-thaw sperm showed significantly (*p* < 0.05) lower Mn-SOD mRNA expression than fresh sperm. Among post-thaw sperm, sperm cryopreserved using 3% sucrose combined with 8% DMSO showed significantly (*p* < 0.05) higher Mn-SOD mRNA expression.

#### 3.6.4. Expression Analysis of GPx mRNA Transcript

Saccharide supplemented cryopreserved sperm showed higher GPx mRNA transcript levels compared to sperm cryopreserved without supplementation of saccharides. However, sperm cryopreserved using 3% sucrose combined with 8% DMSO showed higher mRNA expression of GPx than other types of post-thaw sperm (Figure 9D).

#### 3.6.5. Expression Analysis of GR mRNA Transcript

Results of mRNA abundance of GR in fresh and cryopreserved sperm are given in Figure 9E. Saccharide supplemented cryopreserved sperm showed higher GR mRNA transcript levels compared to sperm cryopreserved without supplementation of saccharides. Sperm cryopreserved using 3% sucrose combined with 8% DMSO had higher GR mRNA expression than sperm cryopreserved using other types of cryoprotectant. However, GR mRNA transcript levels of sperm cryopreserved using 3% sucrose combined with 8% DMSO was not significantly (*p* > 0.05) different from that of sperm cryopreserved using 1% glucose combined with 8% EG.

### 3.7. Expression Analysis of Apopotosis Regulated Gene in Saccharide Supplimented Cryopreserved Sperm

#### 3.7.1. Expression Analysis of Bax mRNA Transcript

**Figure 10:**
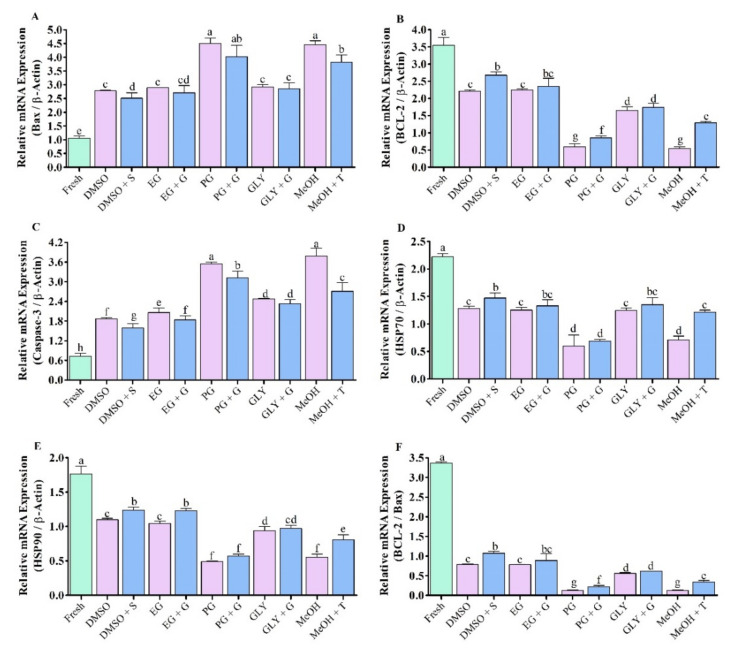
mRNA abundance of antioxidant gene in post-thaw Pacific abalone sperm cryopreserved using saccharides. (**A**) Apoptosis regulator BCL-2 Associated X (Bax), (**B**) B-cell lymphoma 2 (BCL-2), (**C**) Caspase-3, (**D**) Heat shock protein 70 (HSP70), (**E**) Heat shock protein 90 (HSP90), (**F**) BCL-2/Bax ratio in fresh and cryopreserved sperm samples. Expression values of mRNA were normalized against average ΔCT values of control. DMSO: 8% dimethyl sulfoxide, EG: 8% ethylene glycol, PG: 6% propylene glycol, GLY: 2% glycerol, MeOH: 2% methanol, DMSO + S: 8% DMSO combined with 3% sucrose (S), EG + G: 8% EG combined with 1% glucose (G), PG + G: 6% PG combined with 2% glucose (G), GLY + G: 2% GLY combined with 3% glucose (G), MeOH + T: 2% MeOH combined with 4% trehalose (T). Significantly different levels (*p* < 0.05) are denoted by different letters.

Saccharide supplemented cryopreserved sperm showed lower Bax mRNA transcript levels compared to sperm cryopreserved without supplementation of saccharides. Sperm cryopreserved using 3% S + 8% DMSO showed significantly (*p* < 0.05) lower Bax mRNA abundances (Figure 10A). However, sperm cryopreserved using 3% S + 8% DMSO and those cryopreserved using 1% G + 8% EG did not show significant differences in Bax mRNA abundance (Figure 10A).

#### 3.7.2. Expression Analysis of BCL-2 mRNA Transcript

The expression level of BCL-2 was significantly (*p* < 0.05) decreased in sperm cryopreserved using different types of cryoprotectant solution (Figure 10B). Saccharide supplemented cryopreserved sperm showed higher BCL-2 mRNA expression levels compared to sperm cryopreserved without supplementation of saccharides. However, sperm cryopreserved using 3% sucrose combined with 8% DMSO showed improved abundance of BCL-2 mRNA transcript than those cryopreserved using other types of cryoprotectant.

#### 3.7.3. Expression Analysis of Caspase-3 mRNA Transcript

mRNA expression levels of apoptotic gene Caspase-3 in fresh and cryopreserved sperm are given in Figure 10C. Saccharide supplemented cryopreserved sperm showed lower Caspase-3 mRNA expression levels compared to sperm cryopreserved without supplementation of saccharides (Figure 10C). Sperm cryopreserved using 3% sucrose combined with 8% DMSO showed improved abundance of mRNA transcript than other types of post-thaw sperm except for sperm cryopreserved using 1% glucose combined with 8% EG, which did not show a significant difference in abundance of caspase-3 mRNA transcript with those cryopreserved using 3% sucrose combined with 8% DMSO.

#### 3.7.4. Expression Analysis of HSP70 mRNA Transcript

HSP70 mRNA abundance in saccharides supplemented cryopreserved sperm were significantly lower than that of fresh sperm (Figure 10D). However, saccharides supplemented cryopreserved sperm showed higher HSP70 mRNA abundance compared to sperm cryopreserved without supplementation of saccharides (Figure 10D). Sperm cryopreserved using 8% DMSO + 3% S showed higher HSP70 mRNA abundance than those cryopreserved using other types of cryoprotectant (Figure 10D).

#### 3.7.5. Expression Analysis of HSP90 mRNA Transcript

HSP90 mRNA abundance in saccharides supplemented cryopreserved sperm were significantly lower than fresh sperm (Figure 10E). However, saccharides supplemented cryopreserved sperm showed higher HSP90 mRNA abundance compared to sperm cryopreserved without supplementation of saccharides (Figure 10E). Sperm cryopreserved using 8% DMSO + 3% S showed higher HSP90 mRNA abundance than those cryo-preserved using other types of cryoprotectant (Figure 10E). However, HSP90 mRNA abundance of sperm cryopreserved using 8% DMSO + 3% S was not significantly (*p* > 0.05) different from that of sperm cryopreserved using 8% EG + 1% G.

### 3.8. Ratio of BCL-2 to Bax mRNA Expression

Ratios of mRNA expression of BCL-2 to Bax in fresh and cryopreserved sperm are given in Figure 10F. Saccharides supplemented cryopreserved sperm showed higher ratios of mRNA expression of BCL-2 to Bax compared to sperm cryopreserved without supplementation of saccharides. However, sperm cryopreserved using 3% sucrose combined with 8% DMSO had higher ratio of BCL-2 to Bax than sperm cryopreserved using other types of cryoprotectant.

### 3.9. Correlations of Post-Thaw Motility with Oxidative Stress Associated Parameters

Correlations among post-thaw sperm quality parameters, antioxidant activity, lipid peroxidation and DNA integrity are presented in Table 2. ROS production showed moderate negative correlations with antioxidant enzymes (CAT, SOD, and GSH) and moderate positive correlations with MDA content (Table 2). Sperm DNA integrity also showed moderate positive correlations with antioxidant enzymes (Table 2).

### 3.10. Apoptosis Pathway of Saccharide Supplemented Cryopreserved Sperm of Pacific Abalone

**Figure 11:**
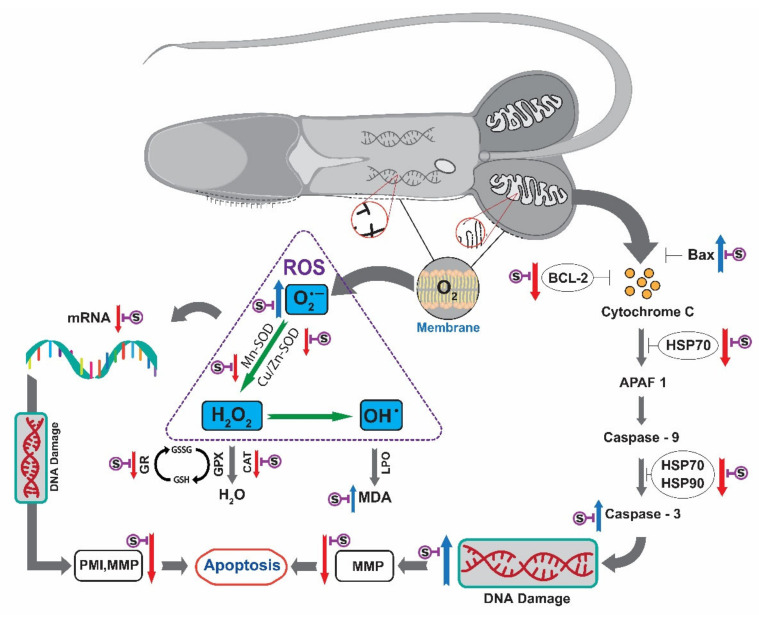
Oxidative stress-oriented apoptosis pathway of cryopreserved sperm of Pacific abalone, *H. discus hannai* and influence of saccharides in the pathway. Cryopreserved sperm showed higher mRNA expression of Bax (apoptosis gene) but lower mRNA expression of BCL-2 (anti-apoptosis gene). Higher mRNA expression of Caspase-3 was found when HSP70 and HSP90 showed lower mRNA expression. Such molecular alterations led to sperm DNA damage by reducing MMP, finally leading to apoptosis. Freeze–thaw process of cryopreservation disrupted the plasma membrane of sperm that produced O_2_ from interspace of membrane and converted to O_2_^•−^. Cryopreserved sperm showed higher production of O_2_^•−^ and MDA when antioxidant enzyme (CAT, SOD, and GSH) showed lower activities and five vital antioxidant genes (CAT, Cu/Zn-SOD, Mn-SOD, GPx, and GR) showed reduced mRNA expression. This disruption of oxidative defense damaged DNA, PMI and MMP, finally leading to apoptosis. However, saccharide supplemented cryopreserved sperm showed improvement against all parameters of apoptosis. The influence of saccharide is marked using a symbol, 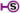 in the apoptosis pathway where appropriate.

An oxidative stress-oriented apoptosis pathway of saccharide supplemented cryopreserved sperm of Pacific abalone is shown in Figure 11. The pathway of post-thaw sperm was adopted based on the findings of enzymatic activity (CAT, SOD, GSH), MDA content, O_2_^•−^, mRNA expression of antioxidant and apoptosis associated gene, PMI, and MMP (Figure 11).

### 3.11. Fertilization and Hatching Rate of Saccharides Supplemented Cryopreserved Sperm

**Figure 12:**
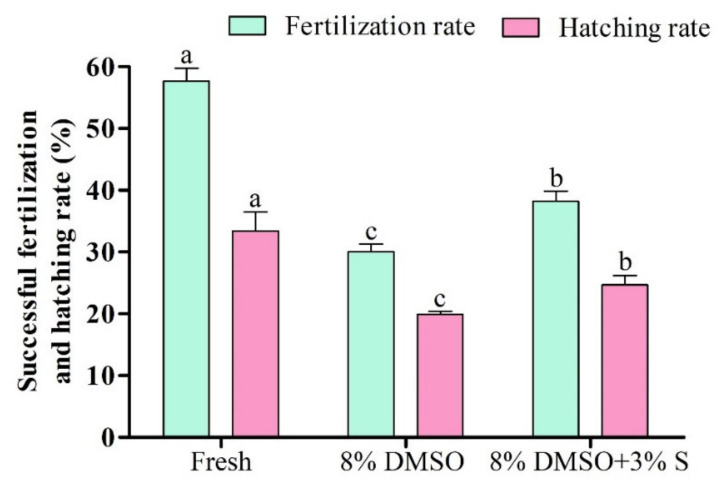
Fertilization and hatching rate of saccharide supplemented cryopreserved sperm of Pacific abalone. DMSO; dimethyl sulfoxide, and S; sucrose. Significantly different levels (*p* < 0.05) are denoted by different letters.

Saccharide supplementation with P-CPA (8% DMSO + 3% S) showed improved fertilization and hatching rates compared to sperm cryopreservation using P-CPA only (Figure 12). However, sperm cryopreserved using 3% sucrose combined with 8% DMSO showed significantly (*p* < 0.05) lower fertilization and hatching rates than fresh sperm.

## 4. Discussion

The goal of the present study was to investigate the antioxidant activity and adopt an oxidative stress-oriented apoptosis pathway in saccharide supplemented cryopreserved sperm of Pacific abalone, *H. discus hannai*. This research group has previously reported sperm cryopreservation technique of Pacific abalone using penetrating cryoprotectant (P-CPA), and antifreeze protein combined with P-CPA [2,3,26]. However, there have not any information about antioxidant status and apoptosis pathway of cryopreserved sperm of this valuable species. Considering the lack of evidence in the previous report, the present study evaluated enzymatic activity (CAT, SOD, and GSH), lipid peroxidation (MDA content), ROS production (superoxide anion: O_2_^•−^), mRNA stability of five vital antioxidant gene (CAT, Cu/Zn-SOD, Mn-SOD, GPx, and GR), and mRNA stability of apoptosis associated gene (Bax, BCL-2, Caspase-3, HSP70, and HSP90) in saccharide supplemented cryopreserved sperm of Pacific abalone.

Saccharides have been previously used to improve the sperm cryopreservation technique of marine invertebrates [30,31,32,33]. In this study, the combination of saccharides with P-CPA improved the post-thaw sperm motility than cryopreservation with P-CPA alone. This is likely because saccharides such as sucrose and glucose stabilize cell membrane phospholipids during the freezing step of cryopreservation [34]. On the other hand, trehalose (a nonreducing disaccharide of glucose) exhibits a defensive role against osmotic effects of cryopreservation process [35,36]. Trehalose also protects intracellular ice crystallization of sperm and protect fertility reduction [36].

The cryopreservation process is responsible for oxidative stress of post-thaw sperm. Oxidative stress involves several factors including excessive ROS production, imbalance antioxidant system, and mitochondrial dysfunction [37,38,39]. These alterations are affected by physical alterations including osmotic stress and temperature variations during the freeze–thaw process of cryopreservation [40]. Cryopreservation can lead to the generation of ROS such as superoxide anion (O_2_^•−^), hydroperoxyl (HOO^•^), and hydroxyl radicals (^•^OH) responsible for sperm structure damage by reducing post-thaw motility, viability, and fertilizing ability [41,42]. The oxidative stress in post-thaw sperm can be evaluated by directly measuring O_2_^•^^−^ production [28,33] or indirectly measuring lipid peroxidation (LPO) or malondialdehyde (MDA) as an oxidative stress marker [9,43]. Saccharides supplemented with P-CPA reduced O_2_^•−^ and MDA levels in post-thaw sperm more than the control without supplementation of saccharides. However, 3% S + 8% DMSO resulted in significantly lower O_2_^•−^ production and MDA level than other types of cryoprotectants. Similar phenomena have been previously reported for post-thaw oyster and salmon sperm cryopreserved using saccharides or vitamins combined with P-CPAs [28,33,44]. The present findings indicate that sucrose supplementation to P-CPA might be responsible for the reduction of O_2_^•−^ production and MDA level. Sandoval-vargas et al. [28] have stated that higher O_2_^•−^ production and increased MDA level might be responsible for sperm plasma membrane damage. The present study revealed similar phenomena, showing that lower MMP of post-thaw sperm were associated with higher O_2_^•−^ and MDA. Excessive production of ROS (O_2_^•−^) and increased level of MDA might be responsible for apoptosis of sperm (Figure 11), which was confirmed by PMI and MMP tests.

The freeze–thaw process can make sperm more susceptible to oxidative stress by changing activities of antioxidant enzymes [33]. Antioxidant enzymes are vital for the defense system of cells by maintaining a suitable ROS level [45]. The intermembrane space of post-thaw sperm can generate excessive O_2_ and produce O_2_^•−^ [46]. Superoxide dismutase (SOD) enzyme can metabolize O_2_^•−^ and produce hydrogen peroxide (H_2_O_2_). Catalase (CAT) and glutathione peroxidase (GPx) can convert H_2_O_2_ into H_2_O and O_2_ [47]. Fenton reaction can convert H_2_O_2_ into ^•^OH and finally increase MDA level by lipid peroxidation (LPO) [46]. Glutathione reductase (GR) controls the supply of reduced glutathione (GSH) by reducing oxidized glutathione (GSSG) to GSH. It plays a vital role in the cellular control of ROS [48,49]. In the present study, cryopreservation reduced activities of antioxidant enzymes and the abundance of mRNAs of five vital antioxidant enzyme genes (CAT, Cu/Zn-SOD, Mn-SOD, GPx, and GR). However, sperm cryopreservation using saccharides improved antioxidant enzyme activities and the abundance of mRNAs of five vital antioxidant enzyme genes in post-thaw sperm. Improved enzymatic activities of antioxidant enzymes in sperm cryopreserved using saccharides have been previously reported for oyster sperm [33], consistent with present findings. To date, except for GPx [50], there has been no information on mRNA expression levels of antioxidant genes in aquatic organisms. Riesco et al. [50] have reported that the mRNA abundance of GPx is significantly decreased in cryopreserved sperm of sole fish, *Solea senegalensis*, compared to that in fresh sperm. However, cryopreserved sperm of mammals has been reported to show reduced mRNA expression of antioxidant gene that can lead to apoptosis of sperm [15,51]. Present findings suggest that the freeze–thaw process can impair antioxidant enzyme activities and lead to apoptosis of sperm (Figure 11). Estudillo et al. [11] reported that sperm cryopreservation can stimulate ROS production and reduce concentrations of antioxidant factors, which are correlated with DNA fragmentation, LPO, and apoptosis of post-thaw sperm.

Intact DNA is essential to provide secured genetic materials to the offspring [52]. Oxidative stress is responsible for sperm DNA fragmentation [53,54]. ROS can oxidize sperm DNA base through MDA of LPO, thus causing a breakdown of double-strand DNA and finally leading to apoptosis [51]. The present study showed that sperm cryopreserved using P-CPA had the highest DNA fragmentation, which correlated with the value of ROS (O_2_^•−^) production. However, saccharides supplemented with P-CPA improved post-thaw sperm DNA integrity more than those cryopreserved with P-CPA alone. It indicates that supplementing saccharides is beneficial for maintaining the stability of post-thaw DNA integrity. Although saccharides cannot penetrate the plasma membrane, they can create an osmotic pressure and induce cell dehydration. It is known that saccharides can interact with plasma membrane phospholipids and increase sperm survivability during the freezing step of cryopreservation [55].

Cryopreservation is responsible for ROS-induced apoptosis in sperm [18,19]. The freezing process can disrupt the mitochondria and alter the abundance of mitochondrial functional genes [56]. These mitochondrial alterations can lead to the release of apoptogenic factor (cytochrome C) from the inner mitochondrial membrane to the cytosol, where cytochrome C can bind to apoptosis protease activating factor-1 (APAF1). APAF1 then triggers Caspase-9 and Caspase-3 and leads to apoptosis of cell [57]. This pathway is mainly regulated by apoptotic (Bax) and anti-apoptotic (BCL-2) genes [58,59,60]. mRNA expression levels of Bax and BCL-2 were quantified in the present study to check the impact of cryopreservation on apoptosis. Results showed upregulated expression of Bax and downregulated expression of BCL-2 in sperm cryopreserved using 3% S + 8% DMSO, resulting in an increase in Bcl-2/Bax ratio. Such an increase of Bcl-2/Bax has been connected to the apoptosis pathway of post-thaw sperm. Caspases are key controllers of the apoptotic pathway in cells [61]. They can induce DNase activity, which is associated with apoptosis and DNA degradation [62]. Caspases are differentiated into initiator and executioner caspases. Caspase-3 is a member of executioner caspases. It is activated after cleaving by initiator caspases [20,61]. During the execution phase of apoptosis, cells undergo changes including nuclear and chromosomal DNA fragmentation, chromosomal condensation, cell shrinkage, and blebbing [63]. Cryopreservation can increase the activation of specific aspartic acid-directed cysteine proteases (caspases) in mammal sperm [20]. Caspase-3 activation has been reported as a partial consequence of DNA fragmentation [64]. The present study supports the previously reported phenomena by showing upregulated mRNA expression of Caspase-3 and higher DNA fragmentation in post-thaw sperm. The present study presented an apoptosis pathway of post-thaw Pacific abalone sperm (Figure 11). To the best of our knowledge, this is the first possible apoptosis pathway reported for cryopreserved sperm of aquatic species. Although similar phenomena have been previously reported from cryopreserved sperm of mammals [15,51], the present study showed higher mRNA abundance of Caspase-3 when HSPs (HSP70 and HSP90) showed lower abundance. Present findings indicate that HSPs might be able to inhibit the overexpression of caspase-3 to prevent the apoptosis of sperm. HSP70 can bind to Bax [65] and direct interact with APAF1, finally prevent apoptosome formation [57]. HSP90 can also inhibit cytochrome C-mediated APAF1 and Caspase-9/Caspase-3 activation [66].

The present study reports correlations among post-thaw sperm quality parameters, antioxidant activity, and lipid peroxidation. Post-thaw quality indicators of sperm showed positive relationships with antioxidant activity. Present findings were consistent with results of Lone et al. [67]. The present findings suggest that improved antioxidant activity and reduced levels of MDA and DNA fragmentation might be responsible for improved post-thaw sperm quality indicators.

Fertilization and hatching capacity ensured the reproduction succession of cryopreserved sperm. In the present study, saccharide (3% sucrose) combined with P-CPA (8% DMSO) improved fertilization and hatching rates more than those cryopreserved without supplementation of saccharides (8% DMSO only). Similar phenomena have been reported previously for cryopreserved greenlip abalone [27] and salmon sperm [28]. This improvement might be due to protective effects of saccharides on sperm quality indicators of Pacific abalone as discussed in previous sections.

## 5. Conclusions

The present study has been adopted an apoptosis pathway for cryopreserved sperm of Pacific abalone and the influence of saccharide in the pathway. This is the first report of apoptosis pathway in cryopreserved sperm of Pacific abalone as well as other organisms. When sperm is cryopreserved, supplementation with saccharides positively influences enzymatic activity, O_2_^•−^ production, apoptosis regulatory gene expression, and fertilization potential of Pacific abalone, *H. discus hannai*. Specifically, 3% sucrose combined with 8% DMSO improved enzymatic activity (CAT, SOD, and GSH), mRNA expression of antioxidant genes (CAT, Cu/Zn-SOD, Mn-SOD, GPx, GR), anti-apoptosis (BCL-2, HSP70, and HSP90) genes and decreased O_2_^•−^ production compared to other examined cryoprotectants. Additionally, 3% sucrose combined with 8% DMSO also showed lower mRNA expression of the apoptosis genes (Bax, and Caspase-3) compared to other examined cryoprotectants. All examined parameters revealed that 3% sucrose combined with 8% DMSO is comparatively more suitable cryoprotectant for sperm cryopreservation of Pacific abalone compared to other cryoprotectants. It might also be useful for molecular conservation of abalone sperm and commercial fertilization in hatcheries.

## Figures and Tables

**Table 1:** List of primers used for qRT-PCR quantification of genes in sperm.

Gene	Primer	Sequence (5′---3′)	Amplicon Length (bp)	GenBank Accession
β-Actin	Sense	CCGTGAAAAGATGACCCAGA	204	AY380809.1
	Antisense	TACGACCGGAAGCGTACAGA		
CAT	Sense	CTGAGAGAGTCGTACATGC	153	OK042347.1
	Antisense	CCTTCTCACCACCTACAGTT		
Cu/Zn-SOD	Sense	GCTGAGAGGTGATTCGGAAG	158	KX302627.1
	Antisense	CACTGGTACAGCCATTGGTG		
Mn-SOD	Sense	TCTCGAGCCCTACATCTCG	208	KX302628.1
	Antisense	GCTAAGCACCTCCCAGAAG		
GPx	Sense	ACAGAAAGTCGCGTGTCAAG	200	GU254066.1
	Antisense	GTCCAGCATAGTCGGTCATG		
GR	Sense	CGTTCGATTACCTGGTGATC	198	OL944707.1
	Antisense	TCACGAACACAAGGAGTACG		
Caspase-3	Sense	ACTTCCTCGTCGCATATGC	171	MT506591.1
	Antisense	CAGGATAATCATGGGCGACT		
Bax	Sense	AGCAAACAACCAGTGGATCG	185	MT501464.1
	Antisense	GCTATCTCTGTCGTGTGTTG		
BCL-2	Sense	AACGAAATGGCTACGTGTGG	175	MT482025.1
	Antisense	GCATGAAATGTTGGATACGCT		
HSP70	Sense	CAGAGAACACAATCTTCGATGC	277	DQ324856.1
	Antisense	CGTTGAGAGTCGTTGAAGTAAG		
HSP90	Sense	AACAGTACATCTGGGAGTCG	216	GU014545.1
	Antisense	CCTCCTTGTCTCTTTCCTTCT		

**Table 2:** Correlations among post-thaw sperm quality parameters, antioxidant activity, DNA integrity, and lipid peroxidation of saccharide supplemented cryopreserved sperm of Pacific abalone.

	Motility	PMI	MMP	O_2_^•−^	DNA Integrity	CAT	SOD	GSH
**PMI**	0.898 **	1						
**MMP**	0.926 **	0.920 **	1					
**O_2_^•−^**	−0.887 **	−0.885 **	−0.881 **	1				
**DNA integrity**	0.889 **	0.884 **	0.845 **	0.799 **	1			
**CAT**	0.878 **	0.895 **	0.892 **	−0.823 **	0.851 **	1		
**SOD**	0.692 **	0.708 **	0.713 **	−0.721 **	0.591 **	0.693 **	1	
**GSH**	0.813 **	0.875 **	0.870 **	−0.850 **	0.756 **	0.811 **	0.763 **	1
**MDA**	−0.836 **	−0.875 **	−0.868 **	0.847 **	−0.843 **	−0.805 **	−0.708 **	−0.854 **

** The correlation values have been calculated using Pearson analysis. Correlation is significant at *p* < 0.01 (two-tailed).

## Data Availability

All data generated in this study are included in this published article.
